# Crystal structure, Hirshfeld surface analysis and inter­action energy and DFT studies of 1-methyl-3-(prop-2-yn-1-yl)-2,3-di­hydro-1*H*-1,3-benzo­diazol-2-one

**DOI:** 10.1107/S2056989019015779

**Published:** 2019-11-29

**Authors:** Asmaa Saber, Mohamed Srhir, Tuncer Hökelek, Joel T. Mague, Noureddine Hamou Ahabchane, Nada Kheira Sebbar, El Mokhtar Essassi

**Affiliations:** a Laboratoire de Chimie Organique Hétérocyclique URAC 21, Pôle de Compétence Pharmacochimie, Av. Ibn Battouta, BP 1014, Faculté des Sciences, Université Mohammed V, Rabat, Morocco; b Department of Physics, Hacettepe University, 06800 Beytepe, Ankara, Turkey; c Department of Chemistry, Tulane University, New Orleans, LA 70118, USA; d Laboratoire de Chimie Appliquée et Environnement, Equipe de Chimie Bioorganique Appliquée, Faculté des Sciences, Université Ibn Zohr, Agadir, Morocco

**Keywords:** crystal structure, benzimidazol-2-one, hydrogen bond, C—H⋯π(ring) inter­action, π-stacking, Hirshfeld surface

## Abstract

In the title mol­ecule, C_11_H_10_N_2_O, the di­hydro­benzimidazol-2-one moiety is essentially planar, with the prop-2-yn-1-yl substituent rotated well out of this plane. In the crystal, C—H_Mthy_⋯π(ring) inter­actions and C—H_Prop_⋯O_Dhyr_ (Mthy = methyl, Prop = prop-2-yn-1-yl and Dhyr = di­hydro) hydrogen bonds form corrugated layers parallel to (10), which are associated through additional C—H_Bnz_⋯O_Dhyr_ (Bnz = benzene) hydrogen bonds and head-to-tail, slipped, π-stacking [centroid-to-centroid distance = 3.7712 (7) Å] inter­actions between di­hydro­benzimidazol-2-one moieties. The Hirshfeld surface analysis of the crystal structure indicates that the most important contributions to the crystal packing are from H⋯H (44.1%), H⋯C/C⋯H (33.5%) and O⋯H/H⋯O (13.4%) inter­actions. Hydrogen-bonding and van der Waals inter­actions are the dominant inter­actions in the crystal packing. Computational chemistry calculations indicate that in the crystal, C—H⋯O hydrogen-bond energies are 46.8 and 32.5 (for C—H_Prop_⋯O_Dhyr_) and 20.2 (for C—H_Bnz_⋯O_Dhyr_) kJ mol^−1^. Density functional theory (DFT) optimized structures at the B3LYP/6–311 G(d,p) level are compared with the experimentally determined mol­ecular structure in the solid state. The HOMO–LUMO behaviour was elucidated to determine the energy gap.

## Abstract 2

The di­hydro­benzimidazol-2-one moiety is essentially planar with the prop-2-yn- 1-yl substituent rotated well out of this plane. In the crystal, C—H⋯π(ring) inter­actions and C—H⋯O hydrogen bonds form corrugated layers parallel to (10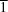), which are associated through additional C—H⋯O hydrogen bonds and head-to-tail, slipped, π-stacking inter­actions between di­hydro­benzimidazol-2-one moieties

## Chemical context   

Benzimidazole is an aromatic heterocyclic organic compound that plays an important role in medicinal chemistry and pharmacology. The most prominent benzimidazole moiety present in nature is *N*-ribosyl-di­methyl­benzimidazole and it serves as the axial ligand for cobalt in vitamin B12 (Walia *et al.*, 2011 ▸). Benzimidazole derivatives possess many biological activities such as anti-microbial, anti-fungal, anti-histaminic, anti-inflammatory, anti-viral, anti-oxidant, anti-cancer and anti-ulcerative (Farukh & Mubashira, 2009 ▸; Ayhan-Kılcıgil *et al.*, 2007 ▸; Soderlind *et al.*, 1999 ▸; Luo *et al.*, 2011 ▸; Navarrete-Vázquez *et al.*, 2011 ▸). They are considered to be an important moiety for the development of mol­ecules of pharmaceutical inter­est (Mondieig *et al.*, 2013 ▸; Lakhrissi *et al.*, 2008 ▸). As a continuation of our research on the development of N-substituted benzimidazole derivatives and the evaluation of their potential pharmacological activities (Saber *et al.*, 2018*a* ▸,*b* ▸, 2020 ▸; Ouzidan *et al.*, 2011 ▸), we have studied the alkyl­ation reaction of iodo­methane with 1-(prop-2-yn­yl)-1*H*-benzoimidazol-2(3*H*)-one in the presence of tetra-*n*-butyl­ammonium bromide as catalyst and potassium carbonate as base, to give the title compound, **I** in good yield. We report herein on its synthesis, the mol­ecular and crystal structures along with the Hirshfeld surface analysis and the inter­molecular inter­action energies and the density functional theory (DFT) computational calculations carried out at the B3LYP/6–311 G(d,p) level for comparison with the experimentally determined mol­ecular structure in the solid state.
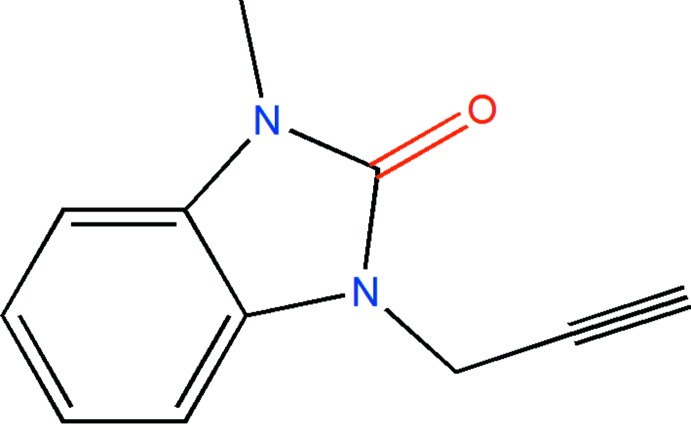


## Structural commentary   

**Figure 1:**
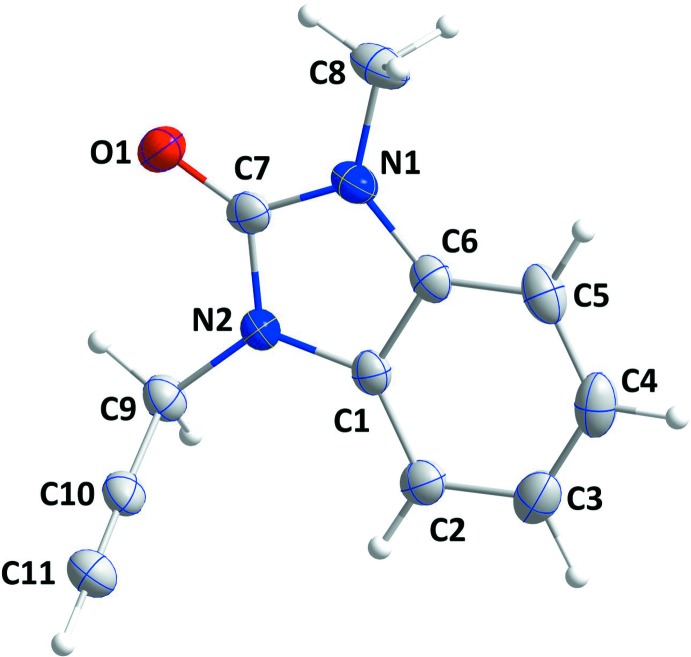
The mol­ecular structure of the title compound with the atom-numbering scheme. Displacement ellipsoids are drawn at the 50% probability level.

In the title compound, the di­hydro­benzimidazol-2-one moiety is planar to within 0.0160 (8) Å (r.m.s. deviation = 0.0082) with atom C7 deviating the most from the mean plane and a prop-2-yn-1-yl substituent rotated well out of this plane as shown by the C1—N2—C9—C10 torsion angle of 62.16 (13)° (Fig. 1 ▸).

## Supra­molecular features   

**Figure 2:**
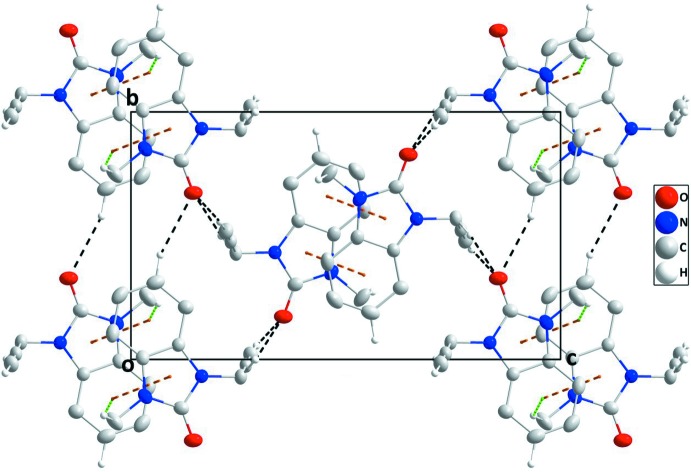
A partial packing diagram viewed along the *a*-axis direction with C—H⋯O hydrogen bonds, C—H⋯π(ring) and π-stacking inter­actions shown, respectively, by black, green and orange dashed lines.

In the crystal, inversion dimers are formed by pairs of C—H_Mthy_⋯*Cg*1^i^ inter­actions [Mthy = methyl; symmetry code: (i) − *x*, 1 − *y*, 1 − *z*; *Cg*1 is the centroid of the benzene (*A*; C1–C6), ring]; which are connected along the *b*-axis direction by C—H_Bnz_⋯O_Dhyr_ hydrogen bonds (Bnz = benzene and Dhyr = di­hydro) and along the *a*-axis direction at *ca* 90° to this and parallel to (10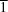) by inversion-related C—H_Prop_⋯O_Dhyr_ hydrogen bonds (Table 1 ▸). The resulting corrugated layers are parallel to (10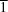) and are connected in pairs by slipped, head-to-tail π-stacking inter­actions between the di­hydro­benzimidazol-2-one moieties, [*Cg*2⋯*Cg*1^ii^ = 3.7712 (7) Å, dihedral angle = 0.96 (6)°; symmetry code: (ii) 1 – *x*, 1 – *y*, 1 – *z*; *Cg*1 and *Cg*2 are the centroids of rings *A* and *B* (N1/N2/C1/C6/C7) and C—H_Prop_⋯O_Dhyr_ (Prop = prop-2-yn-1-yl) hydrogen bonds (Table 1 ▸, Figs. 2 ▸ and 3 ▸).

## Hirshfeld surface analysis   

**Figure 4:**
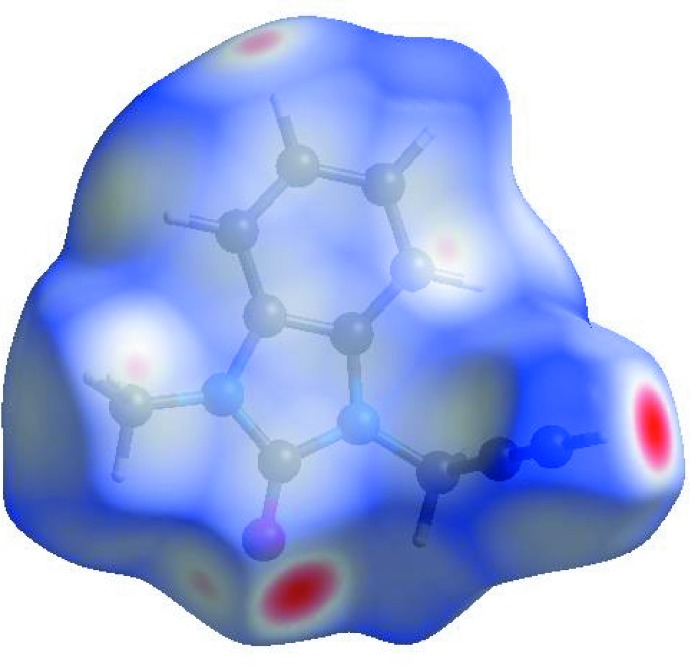
View of the three-dimensional Hirshfeld surface of the title compound plotted over *d*_norm_ in the range −0.3997 to 1.3219 a.u.

**Figure 5:**
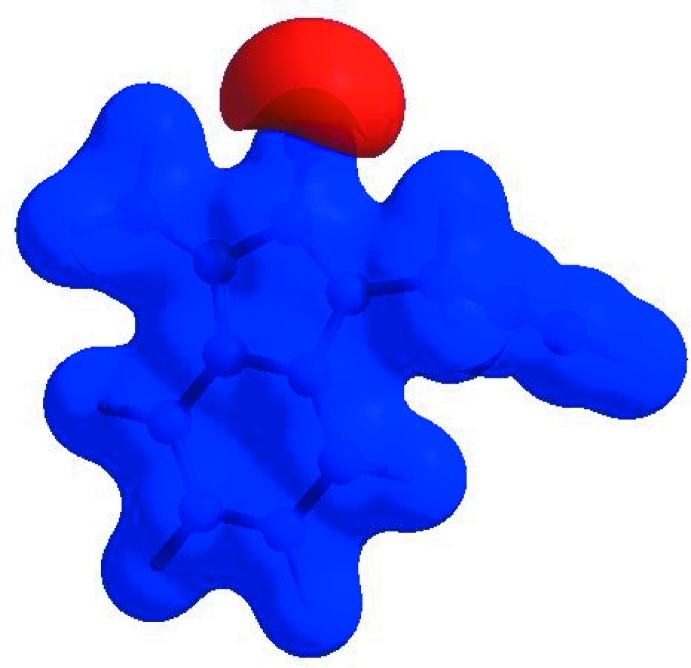
View of the three-dimensional Hirshfeld surface of the title compound plotted over electrostatic potential energy in the range −0.0500 to 0.0500 a.u. using the STO-3 G basis set at the Hartree–Fock level of theory. Hydrogen-bond donors and acceptors are shown as blue and red regions around the atoms corresponding to positive and negative potentials, respectively.

**Figure 6:**
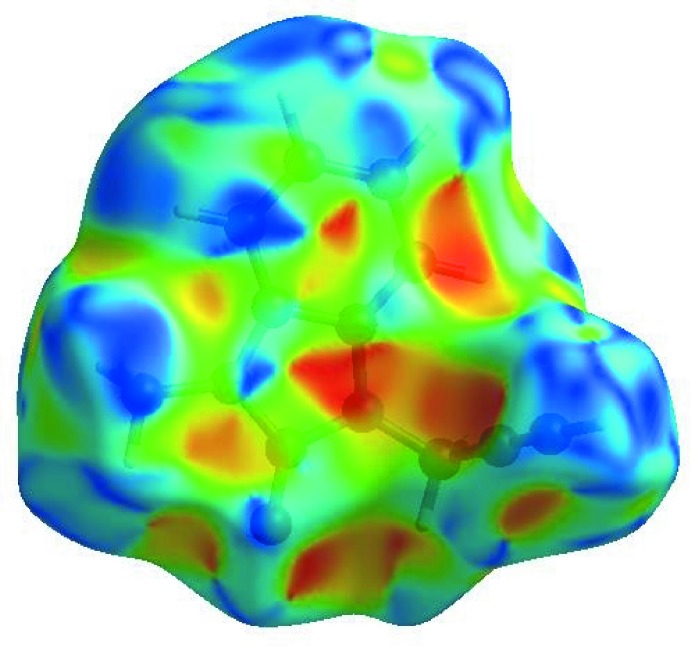
Hirshfeld surface of the title compound plotted over shape-index.

In order to visualize the inter­molecular inter­actions in the crystal of the title compound, a Hirshfeld surface (HS) analysis (Hirshfeld, 1977 ▸; Spackman & Jayatilaka, 2009 ▸) was carried out using *Crystal Explorer 17.5* (Turner *et al.*, 2017 ▸). In the HS plotted over *d*_norm_ (Fig. 4 ▸), the white surface indicates contacts with distances equal to the sum of van der Waals radii, and the red and blue colours indicate distances shorter (in close contact) or longer (distant contact) than the van der Waals radii, respectively (Venkatesan *et al.*, 2016 ▸). The bright-red spots appearing near O1 and the hydrogen atom H11 indicate their roles as the donors and/or acceptors, respectively; they also appear as blue and red regions corresponding to positive and negative potentials on the HS mapped over electrostatic potential (Spackman *et al.*, 2008 ▸; Jayatilaka *et al.*, 2005 ▸) as shown in Fig. 5 ▸. The blue regions indicate positive electrostatic potential (hydrogen-bond donors), while the red regions indicate negative electrostatic potential (hydrogen-bond acceptors). The shape-index of the HS is a tool to visualize π–π stacking by the presence of adjacent red and blue triangles; if there are no adjacent red and/or blue triangles, then there are no π–π inter­actions. Fig. 6 ▸ clearly suggests that there are π– π inter­actions in (I).

**Figure 7:**
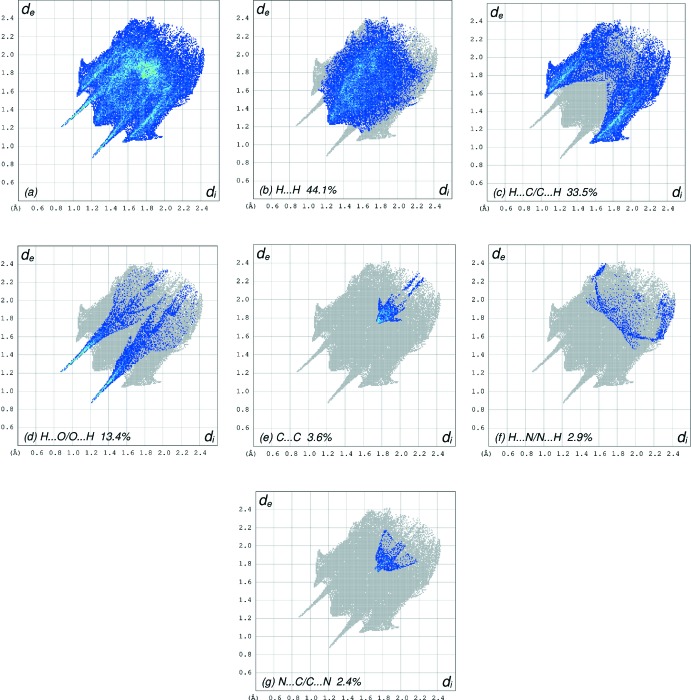
The full two-dimensional fingerprint plots for the title compound, showing (*a*) all inter­actions, and delineated into (*b*) H⋯H, (*c*) H⋯C/C⋯H, (*d*) H⋯O/O⋯H, (*e*) C⋯C, (*f*) H⋯N/N⋯H and (*g*) N⋯C/C⋯N inter­actions. The *d*_i_ and *d*_e_ values are the closest inter­nal and external distances (in Å) from given points on the Hirshfeld surface contacts.

The overall two-dimensional fingerprint plot, Fig. 7 ▸ *a*, and those delineated into H⋯H, H⋯C/C⋯H, H⋯O/O ⋯ H, C⋯C, H⋯N/N⋯H and N⋯C/C⋯N contacts (McKinnon *et al.*, 2007 ▸) are illustrated in Fig. 7 ▸ *b*–*g*, respectively, together with their relative contributions to the Hirshfeld surface. The most important inter­action is H⋯H contributing 44.1% to the overall crystal packing, which is reflected in Fig. 7 ▸ *b* as widely scattered points of high density due to the large hydrogen content of the mol­ecule with the tip at *d*_e_ = *d*_i_ = 1.22 Å. The presence of C—H⋯π inter­actions gives rise to pairs of characteristic wings in the fingerprint plot delineated into H⋯C/C⋯H contacts, Fig. 7 ▸ *c.*, contributing 33.5% to the HS (Table 2 ▸); these are viewed as pairs of spikes with the tips at *d*_e_ + *d*_i_ = 2.56 Å. The pair of wings in Fig. 7 ▸ *d* has a symmetrical distribution of points with the edges at *d*_e_ + *d*_i_ = 2.09 Å arising from the H⋯O/O⋯H contacts (13.4% contribution). The C⋯C contacts, Fig. 7 ▸ *e*, have an arrow-shaped distribution of points with the tip at *d*_e_ = *d*_i_ = 1.75 Å. The H⋯N/N⋯N contacts, contributing 2.9% to the overall crystal packing, are depicted in Fig. 7 ▸ *f* as widely scattered points. Finally, the N⋯C/C⋯N inter­actions, contributing 2.4% to the overall crystal packing, are shown in Fig. 7 ▸ *g* as tiny characteristic wings with the tips at *d*_e_ + *d*_i_ = 3.45 Å.

**Figure 8:**
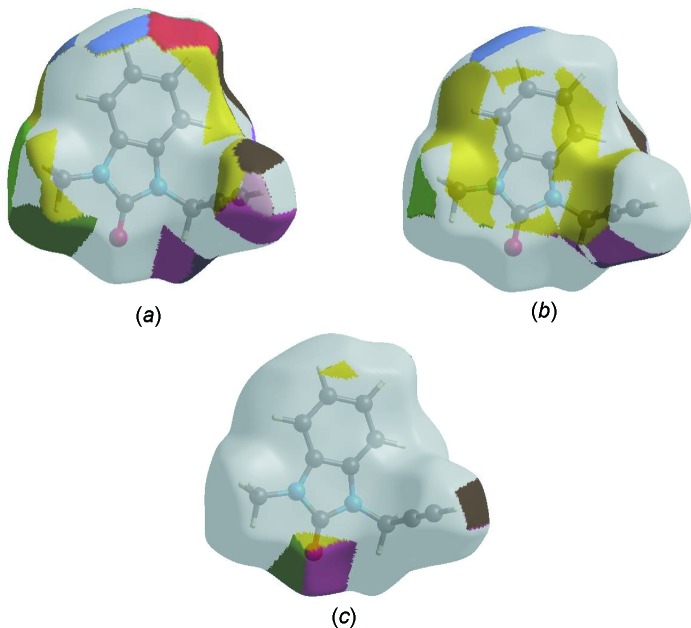
The Hirshfeld surface representations with the function *d*_norm_ plotted onto the surface for (*a*) H⋯H, (*b*) H⋯C/C⋯H and (*c*) H⋯O/O⋯H inter­actions.

The Hirshfeld surface representations with the function *d*_norm_ plotted onto the surface are shown for the H⋯H, H⋯C/C⋯H and H⋯O/O⋯H inter­actions in Fig. 8 ▸ *a*–*c*, respectively.

The Hirshfeld surface analysis confirms the importance of H-atom contacts in establishing the packing. The large number of H⋯H, H⋯C/C⋯H and H⋯ O/O⋯H inter­actions suggest that van der Waals inter­actions and hydrogen bonding play the major roles in the crystal packing (Hathwar *et al.*, 2015 ▸).

## Inter­action energy calculations   

The inter­molecular inter­action energies were calculated using the CE–B3LYP/6–31G(d,p) energy model available in *CrystalExplorer17.5* (Turner *et al.*, 2017 ▸), where a cluster of mol­ecules is generated by applying crystallographic symmetry operations with respect to a selected central mol­ecule within the default radius of 3.8 Å (Turner *et al.*, 2014 ▸). The total inter­molecular energy (*E*_tot_) is the sum of electrostatic (*E*_ele_), polarization (*E*_pol_), dispersion (*E*_dis_) and exchange-repulsion (*E*_rep_) energies (Turner *et al.*, 2015 ▸) with scale factors of 1.057, 0.740, 0.871 and 0.618, respectively (Mackenzie *et al.*, 2017 ▸). Hydrogen-bonding inter­action energies (in kJ mol^−1^) were calculated to be −17.4 (*E*_ele_), −3.5 (*E*_pol_), −62.6 (*E*_dis_), 46.5 (*E*_rep_) and −46.8 (*E*_tot_) for C11—H11⋯O1, −12.4 (*E*_ele_), −1.9 (*E*_pol_), −41.6 (*E*_dis_), 29.6 (*E*_rep_) and −32.5 (*E*_tot_) for C9—H9*B*⋯O1 and −13.7 (*E*_ele_), −3.7 (*E*_pol_), −15.5 (*E*_dis_), 17.0 (*E*_rep_) and −20.2 (*E*_tot_) for C3—H3⋯O1.

## DFT calculations   

**Figure 9:**
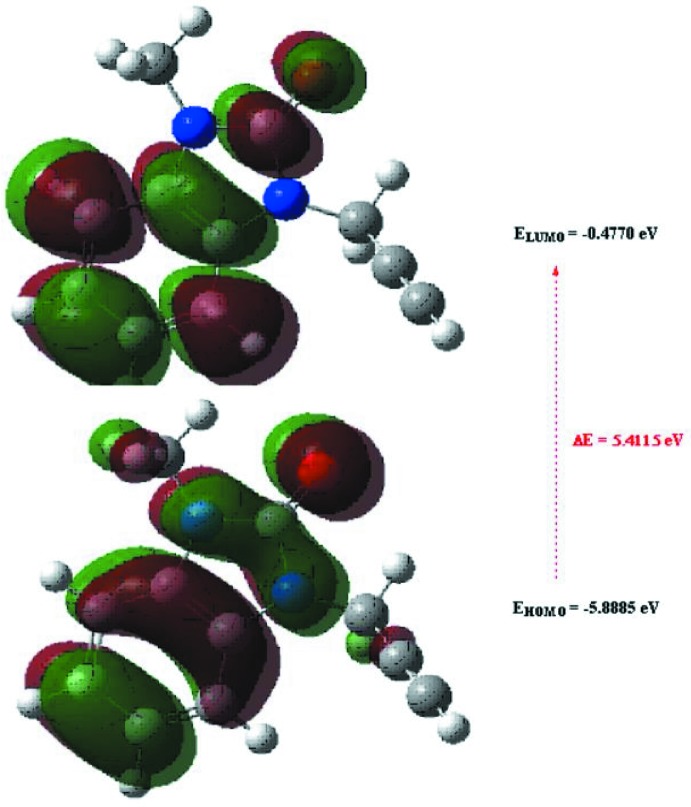
The energy band gap of the title compound.

The optimized structure of the title compound in the gas phase was generated theoretically *via* density functional theory (DFT) using the standard B3LYP functional and 6–311 G(d,p) basis-set calculations (Becke, 1993 ▸) as implemented in *GAUSSIAN 09* (Frisch *et al.*, 2009 ▸). The theoretical and experimental results are in good agreement (Table 3 ▸). The highest-occupied mol­ecular orbital (HOMO), acting as an electron donor, and the lowest-unoccupied mol­ecular orbital (LUMO), acting as an electron acceptor, are very important parameters for quantum chemistry. When the energy gap is small, the mol­ecule is highly polarizable and has high chemical reactivity. The DFT calculations provide some important information on the reactivity and site selectivity of the mol­ecular framework. *E*_HOMO_ and *E*_LUMO_ clarify the inevitable charge-exchange collaboration inside the studied material and are given in Table 4 ▸ along with the electronegativity (χ), hardness (η), potential (μ), electrophilicity (ω) and softness (*σ*). The significance of η and *σ* is for the evaluation of both the reactivity and stability. The electron transition from the HOMO to the LUMO energy level is shown in Fig. 9 ▸. The HOMO and LUMO are localized in the plane extending from the whole 1-methyl-3-(prop-2-yn-1-yl)-2,3-di­hydro-1*H*-1,3-benzo­diazol-2-one ring. The energy band gap [Δ*E* = *E*_LUMO_ − *E*_HOMO_] of the mol­ecule is about 5.4115 eV, and the frontier mol­ecular orbital energies, *E*_HOMO_ and *E*_LUMO_ are −5.8885 and −0.4770 eV, respectively.

## Database survey   

The syntheses of several N-substituted benzimidazol-2-one analogues have been reported (Saber *et al.*, 2018*a* ▸,*b* ▸; 2020 ▸; Belaziz *et al.*, 2012 ▸; Bouayad *et al.*, 2015 ▸; Belaziz *et al.*, 2013 ▸). In a search of the Cambridge Crystallographic Database (CSD; Version 5.40, update of September 2019; Groom *et al.*, 2016 ▸) using benzimidazol-2-one with an exocyclic carbon atom bound to each nitro­gen generated 94 hits. In these, the bicyclic ring system is either planar, has a slight twist end-to-end, or, in the cases where the exocyclic substituents form a ring, has a very shallow bowl shape.
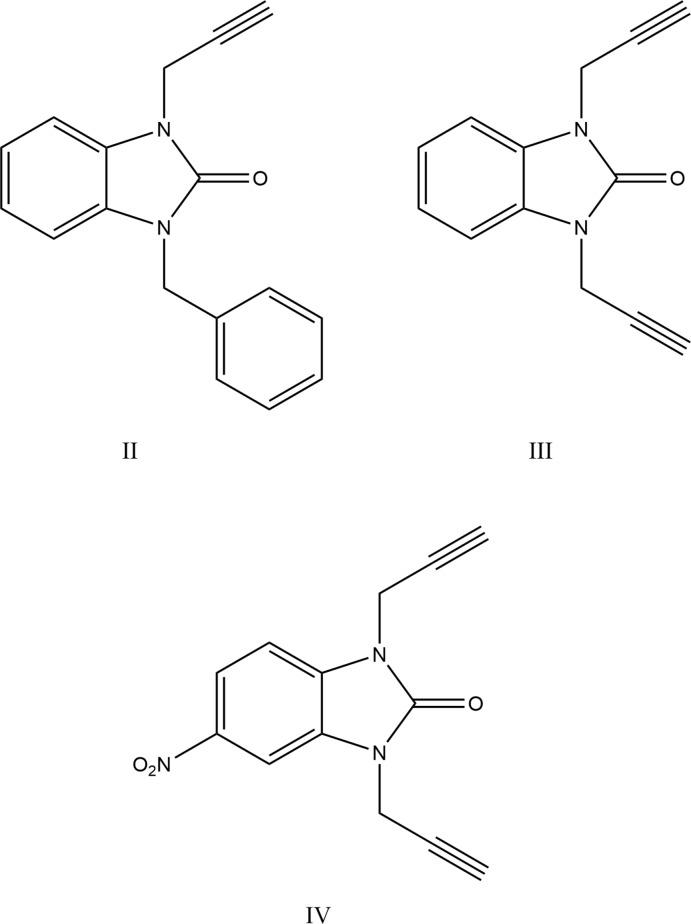


The closest examples to the title compound, **I**, are **II** (HISFUN; Saber *et al.*, 2018*b* ▸), **III** (URAQAG; Ouzidan *et al.*, 2011*a* ▸) and **IV** (AGAXOX; Kandri Rodi *et al.*, 2013 ▸). In the title compound, the C—N bonds to the exocyclic groups are 1.4526 (14) and 1.4545 (19) Å while in **II**–**IV** the corresponding distances range from 1.445 (3) to 1.4632 (11) Å, and so are quite comparable. The exocyclic groups in **I** are in an *anti*-arrangement with the prop-2-yn-1-yl group rotated by 62.16 (13)° out of the plane of the bicyclic moiety (as measured by the C1—N2—C9—C10 torsion angle). In the other three, these substituents are also *anti* and in **II** the corresponding torsion angle is 73.46 (18)° while in **III** they are 82.58 (15) and 74.31 (14)°. In **IV** the torsion angles are 106.0 (3) and 113.4 (3)° indicating a rotation in the opposite direction from the first three.

## Synthesis and crystallization   

To a mixture of 1-(prop-2-yn­yl)-1*H*-benzimidazol-2(3*H*)-one (3.61 mmol), iodo­methane (6.73 mmol) and potassium carbonate (6.24 mmol) in DMF (15 ml) was added a catalytic amount of tetra-*n*-butyl­ammonium bromide (0.37 mmol). The mixture was stirred for 24 h. The solid material was removed by filtration and the solvent evaporated under vacuum. The solid product was purified by recrystallization from ethanol to afford colorless crystals (yield: in 82%).

## Refinement   

Crystal data, data collection and structure refinement details are summarized in Table 5 ▸. Hydrogen atoms were located in a difference Fourier map and refined freely.

## Supplementary Material

Crystal structure: contains datablock(s) I, global. DOI: 10.1107/S2056989019015779/lh5936sup1.cif

Structure factors: contains datablock(s) I. DOI: 10.1107/S2056989019015779/lh5936Isup2.hkl

Supporting information file. DOI: 10.1107/S2056989019015779/lh5936Isup3.cdx

Supporting information file. DOI: 10.1107/S2056989019015779/lh5936Isup4.cml

CCDC reference: 1967468

Additional supporting information:  crystallographic information; 3D view; checkCIF report

## Figures and Tables

**Figure 3:**
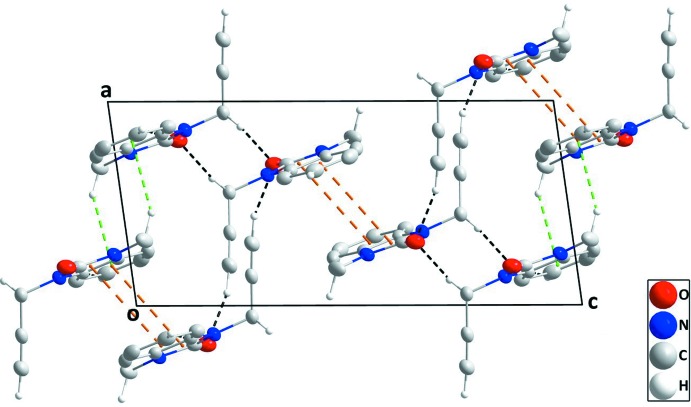
A partial packing diagram viewed along the *b*-axis direction with inter­molecular inter­actions depicted as in Fig. 2 ▸.

**Table 1:** Hydrogen-bond geometry (Å, °) *Cg*1 is the centroid of the C1–C6 benzene ring.

*D*—H⋯*A*	*D*—H	H⋯*A*	*D*⋯*A*	*D*—H⋯*A*
C3—H3⋯O1^ix^	1.005 (15)	2.566 (15)	3.4885 (15)	152.6 (11)
C8—H8*C*⋯*Cg*1^v^	1.004 (16)	2.626 (15)	3.5413 (13)	151.1 (12)
C9—H9*B*⋯O1^vi^	0.978 (15)	2.347 (15)	3.3198 (14)	172.9 (12)
C11—H11⋯O1^vii^	1.010 (15)	2.181 (15)	3.1569 (15)	162.1 (12)

Symmetry codes: (v) 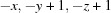; (vi) 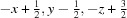; (vii) 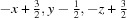; (ix) 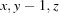.

**Table 2:** Selected interatomic distances (Å)

O1⋯H9*A*	2.491 (14)	C11⋯O1^vii^	3.1569 (15)
O1⋯H3^i^	2.566 (15)	C2⋯H8*A* ^iv^	2.82 (2)
O1⋯H8*B*	2.516 (19)	C3⋯H8*C* ^v^	2.859 (15)
O1⋯H9*B* ^ii^	2.346 (14)	C3⋯H8*A* ^iv^	2.92 (2)
O1⋯H11^iii^	2.181 (15)	C4⋯H8*C* ^v^	2.810 (15)
C2⋯C10	3.3889 (16)	C5⋯H8*C* ^v^	2.935 (15)
C3⋯C8^iv^	3.5335 (17)	C8⋯H5	2.983 (14)
C4⋯C8^v^	3.4947 (17)	C9⋯H2	2.975 (14)
C4⋯C7^iv^	3.5437 (16)	C10⋯H4^viii^	2.976 (15)
C5⋯C8^v^	3.5884 (17)	C11⋯H5^iv^	2.865 (15)
C6⋯C6^iv^	3.5349 (14)	C11⋯H4^viii^	2.705 (15)
C9⋯O1^vi^	3.3198 (14)		

Symmetry codes: (i) 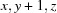; (ii) 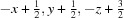; (iii) 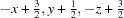; (iv) 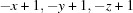; (v) 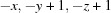; (vi) 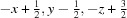; (vii) 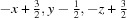; (viii) 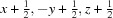.

**Table 3:** Comparison of the selected (X-ray and DFT) geometric data (Å, °)

Bonds/angles	X-ray	B3LYP/6–311 G(d,p)
O1—C7	1.2281 (13)	1.24660
N1—C7	1.3735 (14)	1.39764
N1—C6	1.3874 (15)	1.40100
N1—C8	1.4526 (14)	1.45375
N2—C7	1.3807 (14)	1.40268
N2—C1	1.3910 (13)	1.40222
N2—C9	1.4545 (14)	1.46036
C7—N1—C6	110.19 (9)	110.10303
C7—N1—C8	124.14 (10)	122.94288
C6—N1—C8	125.66 (10)	126.95366
C7—N2—C1	110.16 (9)	110.18664
C7—N2—C9	123.55 (9)	122.02491
C1—N2—C9	126.00 (9)	126.78733
C2—C1—N2	131.64 (10)	132.00719

**Table 4:** Calculated energies for the title compound

Mol­ecular Energy (a.u.) (eV)	
Total Energy *TE* (eV)	−16594.1662
*E*_HOMO_ (eV)	−5.8885
*E*_LUMO_ (eV)	−0.4770
Energy gap, *ΔE* (eV)	5.4115
Dipole moment, *μ* (Debye)	2.8313
Ionization potential, *I* (eV)	5.8885
Electron affinity, *A*	2.6040
Electro negativity, *χ*	0.31828
Hardness, *η*	2.7058
Electrophilicity index, *ω*	1.8719
Softness, *σ*	0.3696
Fraction of electron transferred, *ΔN*	0.7054

**Table 5:** Experimental details

Crystal data	
Chemical formula	C_11_H_10_N_2_O
*M*_r_	186.21
Crystal system, space group	Monoclinic, *P*2_1_/*n*
Temperature (K)	150
*a*, *b*, *c* (Å)	7.1507 (3), 8.8177 (4), 15.4602 (7)
β (°)	97.914 (2)
*V* (Å^3^)	965.52 (7)
*Z*	4
Radiation type	Cu *K*α
μ (mm^−1^)	0.68
Crystal size (mm)	0.32 × 0.31 × 0.12

Data collection	
Diffractometer	Bruker D8 VENTURE PHOTON 100 CMOS
Absorption correction	Multi-scan (*SADABS*; Krause *et al.*, 2015 ▸)
*T*_min_, *T*_max_	0.83, 0.92
No. of measured, independent and observed [*I* > 2σ(*I*)] reflections	6896, 1812, 1679
*R*_int_	0.030
(sin θ/λ)_max_ (Å^−1^)	0.610

Refinement	
*R*[*F* ^2^ > 2σ(*F* ^2^)], *wR*(*F* ^2^), *S*	0.033, 0.086, 1.06
No. of reflections	1812
No. of parameters	168
H-atom treatment	All H-atom parameters refined
Δρ_max_, Δρ_min_ (e Å^−3^)	0.18, −0.19

Computer programs: *APEX3* and *SAINT* (Bruker, 2016 ▸), *SHELXT* (Sheldrick, 2015*a* ▸), *SHELXL2018* (Sheldrick, 2015*b* ▸), *DIAMOND* (Brandenburg & Putz, 2012 ▸) and *SHELXTL* (Sheldrick, 2008 ▸).

## supplementary crystallographic information

### Crystal data

**Table:**

C_11_H_10_N_2_O	*F*(000) = 392
*M**_r_* = 186.21	*D*_x_ = 1.281 Mg m^−^^3^
Monoclinic, *P*2_1_/*n*	Cu *K*α radiation, λ = 1.54178 Å
*a* = 7.1507 (3) Å	Cell parameters from 5848 reflections
*b* = 8.8177 (4) Å	θ = 5.8–70.1°
*c* = 15.4602 (7) Å	µ = 0.68 mm^−^^1^
β = 97.914 (2)°	*T* = 150 K
*V* = 965.52 (7) Å^3^	Plate, colourless
*Z* = 4	0.32 × 0.31 × 0.12 mm

### Data collection

**Table:**

Bruker D8 VENTURE PHOTON 100 CMOS diffractometer	1812 independent reflections
Radiation source: INCOATEC IµS micro-focus source	1679 reflections with *I* > 2σ(*I*)
Mirror monochromator	*R*_int_ = 0.030
Detector resolution: 10.4167 pixels mm^-1^	θ_max_ = 70.1°, θ_min_ = 5.8°
ω scans	*h* = −8→8
Absorption correction: multi-scan (*SADABS*; Krause *et al.*, 2015)	*k* = −10→9
*T*_min_ = 0.83, *T*_max_ = 0.92	*l* = −18→18
6896 measured reflections	

### Refinement

**Table:**

Refinement on *F*^2^	Secondary atom site location: difference Fourier map
Least-squares matrix: full	Hydrogen site location: difference Fourier map
*R*[*F*^2^ > 2σ(*F*^2^)] = 0.033	All H-atom parameters refined
*wR*(*F*^2^) = 0.086	*w* = 1/[σ^2^(*F*_o_^2^) + (0.0402*P*)^2^ + 0.2239*P*] where *P* = (*F*_o_^2^ + 2*F*_c_^2^)/3
*S* = 1.06	(Δ/σ)_max_ < 0.001
1812 reflections	Δρ_max_ = 0.18 e Å^−^^3^
168 parameters	Δρ_min_ = −0.19 e Å^−^^3^
0 restraints	Extinction correction: *SHELXL2018* (Sheldrick, 2015*b*), Fc^*^=kFc[1+0.001xFc^2^λ^3^/sin(2θ)]^-1/4^
Primary atom site location: dual	Extinction coefficient: 0.0100 (12)

### Special details

**Table:**

Geometry. All esds (except the esd in the dihedral angle between two l.s. planes) are estimated using the full covariance matrix. The cell esds are taken into account individually in the estimation of esds in distances, angles and torsion angles; correlations between esds in cell parameters are only used when they are defined by crystal symmetry. An approximate (isotropic) treatment of cell esds is used for estimating esds involving l.s. planes.
Refinement. Refinement of F^2^ against ALL reflections. The weighted R-factor wR and goodness of fit S are based on F^2^, conventional R-factors R are based on F, with F set to zero for negative F^2^. The threshold expression of F^2^ > 2sigma(F^2^) is used only for calculating R-factors(gt) etc. and is not relevant to the choice of reflections for refinement. R-factors based on F^2^ are statistically about twice as large as those based on F, and R- factors based on ALL data will be even larger.

### Fractional atomic coordinates and isotropic or equivalent isotropic displacement parameters (Å^2^)

**Table:**

	*x*	*y*	*z*	*U*_iso_*/*U*_eq_	
O1	0.31019 (11)	0.82725 (9)	0.64517 (6)	0.0345 (2)	
N1	0.24854 (12)	0.64929 (11)	0.53316 (6)	0.0280 (2)	
N2	0.36075 (12)	0.56773 (10)	0.66470 (6)	0.0250 (2)	
C1	0.33940 (14)	0.44082 (11)	0.61080 (7)	0.0235 (2)	
C2	0.37638 (15)	0.28918 (12)	0.62754 (8)	0.0289 (3)	
H2	0.426 (2)	0.2543 (16)	0.6872 (10)	0.039 (4)*	
C3	0.34025 (16)	0.18941 (14)	0.55731 (8)	0.0353 (3)	
H3	0.364 (2)	0.0783 (17)	0.5684 (10)	0.043 (4)*	
C4	0.27117 (17)	0.24106 (15)	0.47421 (8)	0.0378 (3)	
H4	0.246 (2)	0.1678 (17)	0.4255 (10)	0.046 (4)*	
C5	0.23359 (16)	0.39392 (15)	0.45751 (7)	0.0339 (3)	
H5	0.190 (2)	0.4305 (16)	0.3992 (10)	0.042 (4)*	
C6	0.26803 (14)	0.49324 (12)	0.52720 (7)	0.0255 (3)	
C7	0.30715 (14)	0.69684 (12)	0.61712 (7)	0.0260 (2)	
C8	0.17860 (17)	0.75002 (16)	0.46162 (8)	0.0381 (3)	
H8A	0.255 (3)	0.747 (2)	0.4146 (13)	0.076 (6)*	
H8B	0.176 (3)	0.854 (2)	0.4867 (13)	0.072 (5)*	
H8C	0.044 (2)	0.7264 (17)	0.4370 (10)	0.047 (4)*	
C9	0.44506 (16)	0.56993 (13)	0.75585 (7)	0.0283 (3)	
H9A	0.4344 (19)	0.6753 (16)	0.7764 (9)	0.033 (3)*	
H9B	0.376 (2)	0.5012 (16)	0.7898 (9)	0.038 (3)*	
C10	0.64427 (15)	0.52362 (12)	0.76752 (7)	0.0281 (3)	
C11	0.80385 (17)	0.48197 (14)	0.77883 (8)	0.0342 (3)	
H11	0.938 (2)	0.4443 (17)	0.7926 (10)	0.050 (4)*	

### Atomic displacement parameters (Å^2^)

**Table:**

	*U*^11^	*U*^22^	*U*^33^	*U*^12^	*U*^13^	*U*^23^
O1	0.0298 (4)	0.0254 (4)	0.0471 (5)	0.0006 (3)	0.0015 (3)	−0.0012 (3)
N1	0.0239 (4)	0.0309 (5)	0.0277 (5)	−0.0020 (3)	−0.0012 (4)	0.0074 (4)
N2	0.0254 (5)	0.0249 (5)	0.0236 (4)	0.0005 (3)	−0.0007 (3)	−0.0007 (3)
C1	0.0196 (5)	0.0264 (5)	0.0244 (5)	−0.0023 (4)	0.0028 (4)	−0.0014 (4)
C2	0.0249 (5)	0.0281 (6)	0.0337 (6)	−0.0008 (4)	0.0041 (4)	0.0011 (4)
C3	0.0298 (6)	0.0300 (6)	0.0469 (7)	−0.0021 (4)	0.0088 (5)	−0.0072 (5)
C4	0.0339 (6)	0.0427 (7)	0.0384 (6)	−0.0083 (5)	0.0107 (5)	−0.0158 (5)
C5	0.0286 (6)	0.0486 (7)	0.0248 (6)	−0.0089 (5)	0.0048 (4)	−0.0032 (5)
C6	0.0207 (5)	0.0307 (6)	0.0254 (5)	−0.0046 (4)	0.0037 (4)	0.0014 (4)
C7	0.0189 (5)	0.0261 (5)	0.0328 (6)	−0.0007 (4)	0.0025 (4)	0.0025 (4)
C8	0.0294 (6)	0.0450 (7)	0.0380 (7)	−0.0013 (5)	−0.0019 (5)	0.0189 (6)
C9	0.0296 (6)	0.0332 (6)	0.0218 (5)	0.0004 (4)	0.0019 (4)	−0.0014 (4)
C10	0.0333 (6)	0.0293 (5)	0.0206 (5)	−0.0018 (4)	−0.0004 (4)	0.0014 (4)
C11	0.0330 (6)	0.0381 (6)	0.0298 (6)	0.0022 (5)	−0.0016 (4)	0.0030 (5)

### Geometric parameters (Å, º)

**Table:**

O1—C7	1.2281 (13)	C4—C5	1.3915 (19)
N1—C7	1.3735 (14)	C4—H4	0.989 (16)
N1—C6	1.3874 (15)	C5—C6	1.3839 (16)
N1—C8	1.4526 (14)	C5—H5	0.967 (15)
N2—C7	1.3807 (14)	C8—H8A	0.97 (2)
N2—C1	1.3910 (13)	C8—H8B	0.99 (2)
N2—C9	1.4545 (14)	C8—H8C	1.004 (16)
C1—C2	1.3805 (15)	C9—C10	1.4689 (16)
C1—C6	1.4011 (14)	C9—H9A	0.988 (14)
C2—C3	1.3937 (17)	C9—H9B	0.978 (15)
C2—H2	0.991 (15)	C10—C11	1.1885 (17)
C3—C4	1.3883 (19)	C11—H11	1.009 (16)
C3—H3	1.005 (15)		
			
O1···H9A	2.491 (14)	C11···O1^vii^	3.1569 (15)
O1···H3^i^	2.566 (15)	C2···H8A^iv^	2.82 (2)
O1···H8B	2.516 (19)	C3···H8C^v^	2.859 (15)
O1···H9B^ii^	2.346 (14)	C3···H8A^iv^	2.92 (2)
O1···H11^iii^	2.181 (15)	C4···H8C^v^	2.810 (15)
C2···C10	3.3889 (16)	C5···H8C^v^	2.935 (15)
C3···C8^iv^	3.5335 (17)	C8···H5	2.983 (14)
C4···C8^v^	3.4947 (17)	C9···H2	2.975 (14)
C4···C7^iv^	3.5437 (16)	C10···H4^viii^	2.976 (15)
C5···C8^v^	3.5884 (17)	C11···H5^iv^	2.865 (15)
C6···C6^iv^	3.5349 (14)	C11···H4^viii^	2.705 (15)
C9···O1^vi^	3.3198 (14)		
			
C7—N1—C6	110.19 (9)	C5—C6—N1	132.12 (10)
C7—N1—C8	124.14 (10)	C5—C6—C1	120.83 (11)
C6—N1—C8	125.66 (10)	N1—C6—C1	107.04 (9)
C7—N2—C1	110.16 (9)	O1—C7—N1	127.43 (10)
C7—N2—C9	123.55 (9)	O1—C7—N2	126.43 (10)
C1—N2—C9	126.00 (9)	N1—C7—N2	106.14 (9)
C2—C1—N2	131.64 (10)	N1—C8—H8A	112.7 (12)
C2—C1—C6	121.90 (10)	N1—C8—H8B	106.7 (11)
N2—C1—C6	106.45 (9)	H8A—C8—H8B	111.1 (16)
C1—C2—C3	117.07 (11)	N1—C8—H8C	111.9 (9)
C1—C2—H2	120.7 (8)	H8A—C8—H8C	108.5 (15)
C3—C2—H2	122.3 (8)	H8B—C8—H8C	105.7 (13)
C4—C3—C2	121.20 (11)	N2—C9—C10	112.38 (9)
C4—C3—H3	120.5 (9)	N2—C9—H9A	106.5 (8)
C2—C3—H3	118.3 (9)	C10—C9—H9A	109.8 (8)
C3—C4—C5	121.63 (11)	N2—C9—H9B	109.9 (8)
C3—C4—H4	119.6 (9)	C10—C9—H9B	108.2 (8)
C5—C4—H4	118.8 (9)	H9A—C9—H9B	110.1 (11)
C6—C5—C4	117.35 (11)	C11—C10—C9	177.63 (12)
C6—C5—H5	120.9 (8)	C10—C11—H11	176.1 (9)
C4—C5—H5	121.7 (8)		
			
C7—N2—C1—C2	178.91 (11)	C2—C1—C6—C5	−0.69 (15)
C9—N2—C1—C2	4.89 (17)	N2—C1—C6—C5	178.96 (9)
C7—N2—C1—C6	−0.69 (11)	C2—C1—C6—N1	−179.67 (9)
C9—N2—C1—C6	−174.72 (9)	N2—C1—C6—N1	−0.02 (11)
N2—C1—C2—C3	−179.33 (10)	C6—N1—C7—O1	179.51 (10)
C6—C1—C2—C3	0.23 (15)	C8—N1—C7—O1	0.13 (17)
C1—C2—C3—C4	0.37 (16)	C6—N1—C7—N2	−1.13 (11)
C2—C3—C4—C5	−0.54 (18)	C8—N1—C7—N2	179.49 (9)
C3—C4—C5—C6	0.08 (17)	C1—N2—C7—O1	−179.51 (10)
C4—C5—C6—N1	179.20 (11)	C9—N2—C7—O1	−5.31 (16)
C4—C5—C6—C1	0.52 (15)	C1—N2—C7—N1	1.12 (11)
C7—N1—C6—C5	−178.10 (11)	C9—N2—C7—N1	175.32 (9)
C8—N1—C6—C5	1.27 (18)	C7—N2—C9—C10	−111.11 (11)
C7—N1—C6—C1	0.72 (11)	C1—N2—C9—C10	62.16 (13)
C8—N1—C6—C1	−179.91 (9)		

Symmetry codes: (i) *x*, *y*+1, *z*; (ii) −*x*+1/2, *y*+1/2, −*z*+3/2; (iii) −*x*+3/2, *y*+1/2, −*z*+3/2; (iv) −*x*+1, −*y*+1, −*z*+1; (v) −*x*, −*y*+1, −*z*+1; (vi) −*x*+1/2, *y*−1/2, −*z*+3/2; (vii) −*x*+3/2, *y*−1/2, −*z*+3/2; (viii) *x*+1/2, −*y*+1/2, *z*+1/2.

### Hydrogen-bond geometry (Å, º)

*Cg*1 is the centroid of the C1–C6 benzene ring.

**Table:**

*D*—H···*A*	*D*—H	H···*A*	*D*···*A*	*D*—H···*A*
C3—H3···O1^ix^	1.005 (15)	2.566 (15)	3.4885 (15)	152.6 (11)
C8—H8*C*···*Cg*1^v^	1.004 (16)	2.626 (15)	3.5413 (13)	151.1 (12)
C9—H9*B*···O1^vi^	0.978 (15)	2.347 (15)	3.3198 (14)	172.9 (12)
C11—H11···O1^vii^	1.010 (15)	2.181 (15)	3.1569 (15)	162.1 (12)

Symmetry codes: (v) −*x*, −*y*+1, −*z*+1; (vi) −*x*+1/2, *y*−1/2, −*z*+3/2; (vii) −*x*+3/2, *y*−1/2, −*z*+3/2; (ix) *x*, *y*−1, *z*.
